# A Nuclear and Cytoplasmic Characterization of Bovine Oocytes Reveals That Cysteamine Partially Rescues the Embryo Development in a Model of Low Ovarian Reserve

**DOI:** 10.3390/ani11071936

**Published:** 2021-06-29

**Authors:** Valentina Lodde, Alberto Maria Luciano, Giulia Musmeci, Ileana Miclea, Irene Tessaro, Mariella Aru, David F. Albertini, Federica Franciosi

**Affiliations:** 1 Reproductive and Developmental Biology Lab., Dipartimento di Scienze Veterinarie per la Salute la Produzione Animale e la Sicurezza Alimentare ‘Carlo Cantoni’, Università degli Studi di Milano, 20133 Milano, Italy; valentina.lodde@unimi.it (V.L.); alberto.luciano@unimi.it (A.M.L.); giulia.musmeci@studenti.unimi.it (G.M.); ire.tes@libero.it (I.T.); mariellaaru@gmail.com (M.A.); 2 Faculty of Animal Science and Biotechnologies, University of Agricultural Sciences and Veterinary Medicine, 400372 Cluj-Napoca, Romania; ileana.miclea@usamvcluj.ro; 3 Bedford Research Foundation, Bedford, MA 01730, USA; eicjarg@gmail.com

**Keywords:** antral follicle count, early ovarian aging, cow, embryo development, oocyte quality, histone modifications, mitochondria, GSH, cysteamine, gap junctions

## Abstract

**Simple Summary:**

Women’s reproductive performance starts declining in the mid-30s, and by age 40–45, the possibility of becoming pregnant becomes very small. Reproductive aging is a physiological process of fertility decline characterized by a decrease in quality and stockpile of eggs (also called ovarian reserve) in most mammals. However, young individuals too can show an accelerated reproductive aging that similarly results in a low ovarian reserve and hypofertility. This syndrome, called premature ovarian failure (POF), is becoming a relevant problem due to the general tendency to postpone the first pregnancy. In this study, we used bovine ovaries that were classified in two categories, according to the number of follicles visible on the ovarian surface, and analyzed some parameters of egg maturation. We observed that eggs from the ‘aging-like’ ovaries carry several defects that impair maturation. However, one of the parameters was improved upon supplementation with a scavenger of free radicals, providing a proof of concept that in-depth knowledge of the cellular mechanisms is essential to find solutions to everyday-life problems.

**Abstract:**

Decreased oocyte quality is a major determinant of age-associated fertility decline. Similarly, individuals affected by early ovarian aging carry low-quality oocytes. Using an established bovine model of early ovarian aging, we investigated key features of ‘quality’ oocyte maturation, associated with the onset of egg aneuploidy and reproductive aging, such as histone modifications, mitochondria distribution and activity, reduced glutathione (GSH) content, and gap junction functionality. Bovine ovaries were classified according to the antral follicle count (AFC), and the retrieved oocytes were processed immediately or matured in vitro. We observed alterations in several cellular processes, suggesting a multifactorial etiology of the reduced oocyte quality. Furthermore, we performed a rescue experiment for one of the parameters considered. By adding cysteamine to the maturation medium, we experimentally increased the free radical scavenger ability of the ‘low competence’ oocytes and obtained a higher embryo development. Our findings show that adopting culture conditions that counteract the free radicals has a positive impact on the quality of ‘compromised’ oocytes. Specifically, cysteamine treatment seems to be a promising option for treating aging-related deficiencies in embryo development.

## 1. Introduction

With women’s reproductive performance starting to decline in the mid-30s and having extremely small possibilities of becoming pregnant by the age of 40–45 [1,2], female age is one of the most important predictors of reproductive success. A depletion of the follicle reserve and low oocyte quality are critical factors in this fertility decline, which is referred to as reproductive aging [3,4,5]. Despite being a physiological process, reproductive aging has started to pose social and medical challenges due to the recently increased habit to postpone childbearing. It is becoming increasingly common that women around their 40s seek medical support in order to conceive. Furthermore, reproductive aging is associated with a higher incidence of errors in the segregation of the chromosomes, leading to aneuploid eggs, which in turn is responsible for embryo and pregnancy loss and genetic anomalies of the offspring (e.g., Down syndrome) [6,7,8,9].

A distinctive morphological trait of reproductive aging, used to predict the ovarian reserve, is the observation of few antral follicles on the ovary’s surface, referred to as low antral follicle count (AFC) [10,11]. Experimental approaches revealed that the AFC is positively correlated with the extent of the primordial follicle pool [12,13], even though the physiological mechanisms for the association between the two follicular stages have not been fully elucidated. Besides the decrease in the ovarian reserve and oocyte quality, reproductive aging is also associated with a distinctive hormonal profile, characterized by high serum levels of basal follicle-stimulating hormone (FSH) and low anti-Mullerian hormone (AMH) [14,15,16,17,18].

Such features of reproductive aging are not exclusive to women approaching the end of their reproductive life, but are common to several mammals. For instance, they have been described in the cow too [19,20]. Furthermore, some individuals during the reproductive age can show a reduced number of follicles accompanied by suboptimal fertility, diminished ovarian function, and poor oocyte quality, overall impairing the ability to conceive [2,21,22]. This syndrome, which is mainly referred to as early ovarian aging or premature ovarian failure (POF), affects approximately 1–10% of women of reproductive age [23,24,25] and is characterized by accelerated ovarian senescence that results in follicle reduction and hypofertility [26,27,28]. It goes without saying that the tendency to postpone the first pregnancy exacerbates the effects of POF, with patients being diagnosed only when seeking medical assistance to conceive, after failing natural conception.

Two main hypotheses have been proposed to explain the onset of POF: (1) failure to acquire an adequate number of initial primordial follicles, which normally takes place during fetal life, and (2) excessive clearance of primordial follicles, together with the suppressed activation and further development of primordial follicles [24]. The etiology in the majority of POF cases remains idiopathic. However, this condition seems to have a heterogeneous background [28,29], and in the past decade, an increasing number of genes have been associated with POF [23,30,31,32,33,34].

The need for reliable treatments and tests for an early diagnosis has prompted studies in animal models. In particular, mice and cows seemed apt for this purpose. A phenotype similar to POF has been recognized and described in young adult cows [21,22]. Moreover, hypofertility has an important economic impact in dairy farming because it decreases the milk yield and number of calves born while increasing the cost of veterinary services and culling rate [35,36,37]. Currently, up to 50% of dairy cows exhibit abnormal postpartum estrous cycles and ovarian dysfunction resulting in an extended calving-to-first-insemination interval and a decline in conception rates [35,36,37,38].

The aging-like ovary phenotype has been reported in cows by several independent groups, starting from the late 1990s, that described common morphological and functional aspects. Specifically, a consistently low AFC in the ovaries of young adult cows was accompanied by reduced fertility and low rates of embryo development [21,22,39,40,41,42,43]. Further morphological and biochemical characterization underlined a direct relationship between a low AFC and markers of ovarian premature senescence in dairy cows of reproductive age, such as a decreased ovarian size, a decline in the ovarian reserve, a reduction in AMH and estradiol (E_2_) concentrations, and increased progesterone (P_4_) in the follicular fluid in comparison to the age-matched control group of ovaries with normal AFC [43]. Moreover, a compact stroma encapsulating the few healthy medium antral follicles located in aging-like ovaries may contribute to the limited ingrowth of capillaries into the theca, thus isolating the medium antral follicles from the ovarian environment [42]. Finally, aneuploidy rates in oocytes retrieved from aging-like ovaries were greatly increased compared to normal ovaries [44].

Overall, the aging-like bovine ovaries show a phenotype resembling menopausal gonads that is also typical of young women with POF [45] and can therefore be a suitable model to study the causes of low fertility in high-yielding dairy cows, as well as the condition of POF in single-ovulating species. With this in mind, the present study aimed at improving the cellular and molecular characterization of the gametes retrieved from bovine ovaries with low AFC. Specifically, we set out to investigate features connected with the oocyte’s ability to develop as an embryo, called oocyte developmental competence.

Developmental competence relies on a complex set of processes including the coordinated nuclear and cytoplasmic maturation. Nuclear maturation is one of the main events that, through chromosome condensation and segregation, ensure the correct ploidy of the zygote. During maturation, a prophase I-arrested oocyte reaches the metaphase II (MII) stage, marked by the extrusion of the polar body that contains half of the genetic material. Notably, nuclear maturation is accompanied by a significant reorganization of the oocyte cytoplasm, generally identified as cytoplasmic maturation [46,47]. For instance, extensive remodeling and repositioning of intracellular organelles occur, including movements of vesicles, mitochondria, Golgi apparatus, and endoplasmic reticulum [48].

Although not exhaustive, we chose to investigate some parameters that are considered paradigmatic of nuclear and cytoplasmic maturation and have been previously linked to the oocyte developmental competence, such as mitochondria distribution and activity [49,50]; histone modifications [51,52,53], comprising the ones involved in DNA damage [54,55]; reduced glutathione (GSH) content [56,57]; and communications between oocyte and the somatic compartment [58,59], to better understand if and how they might be involved in determining a reduction in the developmental competence in oocytes derived from bovine ‘aging-like’ ovaries. While we observed several alterations in the oocyte maturation process, we also provide evidence that restoring one of the considered parameters was sufficient to improve the embryo development in ‘compromised’ oocytes. Specifically, the supplementation of maturation medium with cysteamine increased the oocyte GSH content and positively impacted the developmental competence.

## 2. Materials and Methods

All the chemicals used in this study were purchased from Sigma-Aldrich Chemical Company, except for those specifically mentioned.

All the experiments were performed at least 3 independent times.

### 2.1. Oocyte Collection and Embryo In Vitro Production

Bovine ovaries were recovered at an abattoir (INALCA SpA, Ospedaletto Lodigiano, Italy) from pubertal dairy cows (4–8 years old) subjected to routine veterinary inspection in accordance with the specific health requirements, as previously reported [60]. Ovaries were isolated and classified into two previously described categories [42], based on the number of medium antral follicles (2–6 mm) visible on the ovarian surface as low AFC (Lo) when displaying <10 follicles or high AFC (Hi) when having ≥10 follicles. Only ovaries isolated from cows having both Lo or both Hi ovaries were used. The presence or absence of a corpus luteum was not taken into account, as previously reported [39,40,41,42,43].

Ovaries were transported at 26 °C, and all the subsequent procedures were performed at 35–38 °C. Cumulus–oocyte complexes (COCs) were retrieved from medium antral follicles with a 16-gauge needle mounted on an aspiration pump (COOK-IVF, Brisbane, QLD, Australia). COCs were washed in TCM-199 supplemented with HEPES buffer 20 mM, 1790 U/L heparin, and 0.4% of bovine serum albumin (BSA) (H-M199) and examined under a stereomicroscope. Only COCs medium-brown in color with five or more complete layers of cumulus cells with oocytes with finely granulated homogeneous ooplasm were used. According to the experimental plan, COCs were either processed immediately to investigate oocytes at the prophase I (so-called germinal vesicle (GV) stage oocytes) or in vitro matured to reach the MII stage. Groups of 15–30 COCs were in vitro matured for 24 h in TCM-199 supplemented with 0.68 mM L-glutamine, 25 mM NaHCO_3_, 0.4% fatty acid free BSA, 0.2 mM sodium pyruvate, and 0.1 IU/mL of recombinant human FSH (Gonal-F, Merck Serono SpA) in humidified air under 5% CO_2_ at 38.5 °C as previously described [61].

In a set of experiments, COCs were in vitro matured with or without 100 µM cysteamine and fertilized as previously described [62]. Briefly, the contents of a straw of cryopreserved bull spermatozoa (CIZ, S. Miniato Pisa, Italy) were thawed, and cells were separated on a 45–90% Percoll gradient. Sperm cells were counted and diluted to a final concentration of 1 × 10^6^ spermatozoa/mL of fertilization medium, which was TALP supplemented with 0.6% (*w*/*v*) BSA fatty acid free, 10 µg/mL heparin, 20 µM penicillamine, 1 µM epinephrine, and 100 µM hypotaurine. COCs were cultured in 300 µL of fertilization medium and incubated for 18 h at 38.5 °C under 5% CO_2_ in humidified air.

After fertilization, presumptive zygotes were cultured as previously reported [42]. Briefly, residual cells and spermatozoa were removed by vortexing for 2 min in 500 µL of synthetic oviduct fluid buffered with 10 mM of HEPES and 5 mM of NaHCO_3_ (SOF wash), rinsed twice, and then transferred in groups of 15–30 in SOF embryo culture medium [63]. The embryo culture SOF was buffered with 25 mM of NaHCO_3_ and supplemented with MEM essential and nonessential amino acids, 0.72 mM of sodium pyruvate, 2.74 mM of myo-inositol, 0.34 mM of sodium citrate, and 5% calf serum (CS). Incubation was performed at 38.5 °C with a humified gas mixture composed of 5% CO_2_, 5% O_2,_ and 90% N_2_. At 48 and 186 h after insemination, cleavage and blastocyst rates were assessed, respectively. Blastocysts were fixed in 60% methanol in PBS, and cell nuclei were dyed and counted as previously described [64,65].

### 2.2. Histone H4 Acetylation at the Lysine Residue K5 and K12

Changes in the acetylation of histone H4 at lysine 5 (acH4K5) and lysine 12 (acH4K12) were investigated in GV and MII stage oocytes by indirect immunofluorescence, using polyclonal anti-AcH4K5 or anti-AcH4K12 antibody (Upstate Biotechnologies, Inc., Lake Placid, NY, USA), as previously described [66,67]. Briefly, COCs were mechanically denuded in 500 mL H-M199 supplemented with 5% CS, and the zona pellucida was removed using 0.2% pronase. After being washed three times in Dulbecco’s phosphate-buffered saline (DPBS) containing 0.1% polyvinyl alcohol (PBS-PVA), the oocytes were fixed in 4% paraformaldehyde in DPBS for 1 h at room temperature. The fixed oocytes were washed three times with PBS-PVA and permeabilized with 0.2% Triton-X 100 in DPBS containing 0.05% Tween 20 (PBS-Tween) for 30 min at room temperature. Nonspecific binding was blocked by incubating the samples in 20% donkey serum, 1% BSA in PBS-Tween for 1 h at room temperature. The samples were then incubated overnight at 4 °C with primary antibody solution 1:250 in PBS-Tween containing 1% BSA. Negative controls were performed by primary antibody omission. After being washed three times in PBS-Tween at room temperature for 10 min each, the oocytes were incubated with TRITC-labeled donkey anti-rabbit antibody (dilution 1:100; Vector Laboratories, Inc., Burlingame, CA, USA). Samples were washed three times in PBS-Tween and mounted on slides in the antifade medium Vecta Shield (Vector Laboratories Inc.) supplemented with 1 µg/mL DAPI for DNA counterstaining. Samples were analyzed using a confocal laser scanning microscope (C1si; Nikon, Tokyo, Japan) with a 60X objective. Digital optical sections were obtained by scanning the samples on the *z*-axis at 0.7 µm of thickness throughout the whole chromatin, as evidenced by DAPI staining. The z-series were then projected to obtain a three-dimensional image. Instrument settings were kept constant for each sample in the red channel, while DAPI settings were kept constant within the same meiotic stage (GV and MII, respectively). Quantification of the relative fluorescence was carried out on digitalized images using NIH ImageJ 1.53e software [68] after background subtraction. Data are expressed as the ratio of the specific acetylated residue fluorescence intensity divided by the DAPI fluorescence intensity.

### 2.3. Phosphorylation of the Histone Variant H2AX (H2AXγ)

Oocytes were simultaneously fixed and extracted in a microtubule-stabilizing buffer containing protease and phosphatase inhibitors as previously described [61,69,70,71,72,73] and then incubated overnight with the primary antibody anti-phospho-histone H2AX (H2AXγ) mouse monoclonal antibody (1:100). The following day, oocytes were washed 4 times for 10 min each in order to remove any surplus antibody and then incubated with the secondary antibody, an Alexa 488-conjugated donkey anti-mouse antibody, diluted 1:500. After another wash cycle, oocytes were mounted on glass slides in DAPI containing antifade medium Vecta Shield, as described above, and examined in confocal microscopy (C1si; Nikon, Tokyo, Japan) with a 60X objective.

The resulting images were imported into Fiji software [74] using the ‘Hyperstack’ option with an adjustment of contrast and brightness, and a protocol was developed based on the study conducted by Cai and coauthors [75]. A composite image or extended in-depth projection (EDH) was created for each oocyte with the help of ‘Image\stacks\Zproject’ and using ‘Maximum intensity’. The region of interest was selected, and the threshold value was manually adjusted using ‘Image\Adjust\Threshold’ for each image depending on the nuclear stage. Then, a particle analysis was performed using ‘Analyze\Analyze Particles’, and the number, area (µm^2^), fluorescence intensity, and integrated density of foci were measured. Based on the extension of their area, H2AXγ-positive foci were divided into 3 categories: small foci (0.021–0.1 µm^2^), medium foci (larger than 0.1–0.3 µm^2^), and large foci (>0.3 µm^2^).

### 2.4. Mitochondria Staining and Image Analysis

Mitochondrial distribution, amount, and activity were analyzed using the fluorescence probes MitoTracker FM Green (MTG), and MitoTracker Orange CMTMRos (MTO) (Invitrogen, Life Technologies, CA, USA). MTG is a fluorescent probe that stains all the mitochondria without taking into account the membrane potential (MMP), while MTO is a lipophilic cationic fluorescent dye that concentrates inside the mitochondria in response to the negative MMP [76].

Procedures for total mitochondria staining with MTG were performed as previously described [65]. Briefly, cumulus cells were removed mechanically from oocytes by vortexing (2 min, 35 Hz) in H-M199 supplemented with 5% CS. Oocytes were incubated for 30 min in pre-equilibrated IVM medium without FSH supplemented with 300 nM MTG and 200 nM MTO (38.5 °C, 5% CO_2_). Oocytes were then washed three times for 3 min in prewarmed PBS containing 0.1% polyvinyl alcohol (PBS-PVA) in the dark. Oocytes were mounted on a prewarmed slide and immediately analyzed under an epifluorescence microscope (Eclipse E600; Nikon). Digital pictures were taken using the fluorescein isothiocyanate (FITC) filter setting to image MTG and the tetramethylrhodamine isothiocyanate (TRITC) filter setting to image MTO, keeping constant exposure times within the same experiment. Images were taken at the equatorial focal plane (i.e., focusing on the largest diameter of the oocytes) and used to analyze the pattern of mitochondria distribution according to morphological criteria [49,50,77] and to conduct quantifications of the relative fluorescence. We observed two classes of MTG distribution (diffuse and cortical ring) and three classes for the MTO (diffuse, cortical ring, and with cytoplasmic aggregates). The fluorescent signal was classified as diffuse when the oocytes showed a homogeneously spread ooplasmic staining, as cortical ring when the staining was restricted to the periphery of the ooplasm, and as aggregated when clumps of various size were observed. In some oocytes, the fluorescence signal was undetectable (11 for MTG and 15 for MTO). The relative fluorescence of MTG and MTO was analyzed using NIH ImageJ 1.53e software [69] and expressed as mean grey values after background subtraction. The oocyte MTO/MTG ratio was calculated as an indicator of the MMP independent of the mitochondrial mass as proposed by [76,78]. Oocytes having an undetectable fluorescent signal were excluded from the quantitative image analyses. Overall, MTG signal was analyzed in 84 oocytes, MTO was analyzed in 75 oocytes, and the MTO/MTG ratio was analyzed in 69 oocytes.

### 2.5. GSH Content

The GSH content was assayed in GV stage and MII stage oocytes that were in vitro matured with or without 100 µM cysteamine as previously described for horse oocytes [79], slightly adapted for bovine oocytes. COCs were stripped free of cumulus cells and washed three times in PBS-PVA. Groups of 5–15 oocytes were transferred at the bottom of an Eppendorf tube, and the excess buffer was removed by aspiration with a narrow bore glass pipette. Samples were snap-frozen and stored at −80 °C until assayed. The GSH content was determined by 5,5′-dithio-bis (2-nitrobenzoic acid) (DTNB)–GSH reductase recycling micro-GSH assay as previously described. Briefly, 50 µL of deionized water was added to each sample that then underwent 3 cycles of freezing and thawing. Freshly prepared standards containing 0.19–200 pmol GSH and samples were assayed in a 96-well microtiter plate. Reaction mixture was prepared with 0.15 mM of DTNB, 0.2 mM of NADPH, and 1.0 U GSH reductase/mL (final concentrations) in 0.1 M phosphate buffer supplemented with 1 mM EDTA, pH 7.8; 0.1 mL/reaction was added directly in the well. The plate was analyzed on a microtiter plate reader (SpectraCount, Packard, Meriden, CT, USA) using 405 nm wavelength with reads repeated every 2 min for 30 min.

### 2.6. Gap Junction Coupling

Intercellular communications between the oocyte and the cumulus cells were assessed by intraooplasmic microinjection of the gap junction (GJ)-permeant fluorescent dye Lucifer yellow (LY), as previously described [80]. A 3% solution of LY in 5 mM lithium chloride was microinjected into the oocyte using a Narishige microinjection apparatus (Narishige Co., Ltd., Tokyo, Japan). The spread of the dye into the surrounding cumulus cells was monitored with an inverted fluorescence microscope (Nikon Diaphot Nikon Corp., Tokyo, Japan) within 10 min. GJs between the oocyte and the cumulus cells were classified as functionally open, partially open, or closed according to the dye spreading to basically the whole cumulus (open), spreading only to a limited portion of the cumulus (partial), or remaining confined to the oocyte (closed).

### 2.7. Statistical Analysis

Statistical analyses were conducted using Prism (GraphPad Software, La Jolla, CA, USA).

Data obtained by quantitative image analyses were first statistically assessed for normal distribution by Shapiro–Wilk test, then the most appropriate statistical test was applied (parametric testing for normally distributed data; nonparametric testing for data that were not normally distributed). Differences in the relative fluorescence of acH4K5, acH4K12, H2AXγ, MTG, and MTO and the number, area, and integrated density of H2AXγ-positive foci were analyzed by Mann–Whitney test (nonparametric). Differences in the mean of the MTG/MTO ratio were analyzed by two-tailed unpaired T-test (parametric). Differences in GSH content, cleavage rate, blastocyst rate, and cell number/blastocyst were analyzed by one-way ANOVA followed by Tukey’s multiple comparison test (parametric). All these data are represented as mean ± standard error of the mean (s.e.m.).

Patterns of mitochondria distribution and GJ classes were analyzed by Fisher’s exact test. Data are presented as percentages of oocytes in each class.

Whenever possible, statistical differences are indicated as * *p* < 0.05, ** *p* < 0.01, *** *p* < 0.001, and **** *p* < 0.0001. However, in some graphs and tables, letters have been used in order to avoid excessive complexity in the interpretation of the figure/table. In such cases, different letters indicate *p* < 0.05.

## 3. Results

### 3.1. Nuclear Maturation

#### 3.1.1. Acetylation of H4 at the Residues K5 and K12

**Figure 1:**
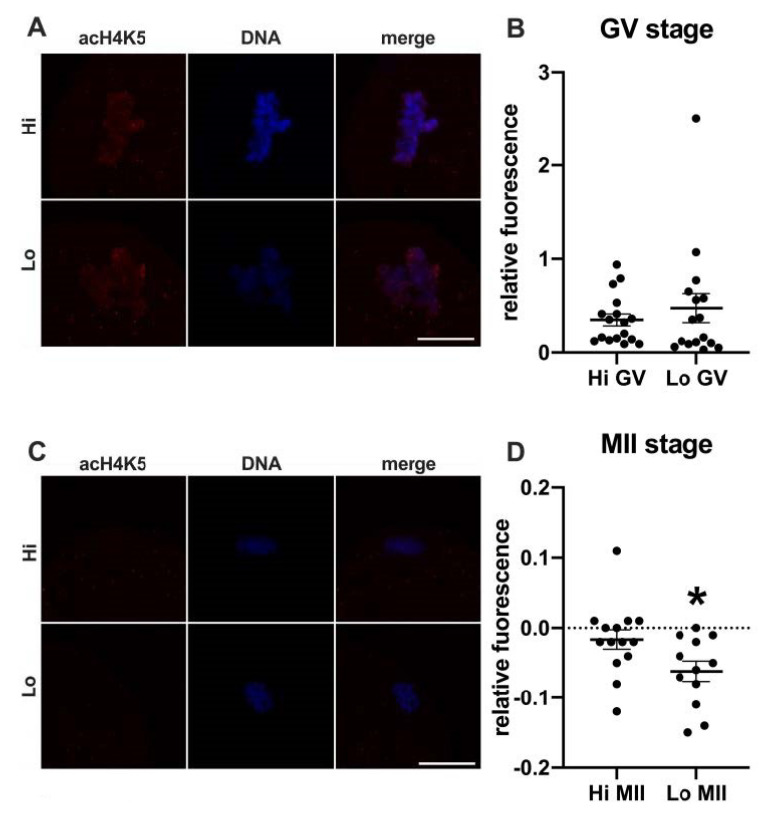
Acetylation of histone H4K5 (acH4K5) in oocytes collected from Hi and Lo ovaries. (**A**,**C**) Representative images of the chromatin of GV oocytes (**A**) and MII oocytes (**C**) stained for acH4K5 (red) and DNA (blue) and merged. Scale bar = 10 μm. (**B**,**D**) The dot blots represent the relative fluorescence of acH4K5 normalized by the DNA signal in GV (**B**) and MII oocytes (**D**). Each dot represents one oocyte. The mean ± s.e.m. is also indicated. * *p* < 0.05, Mann–Whitney test.

**Figure 2:**
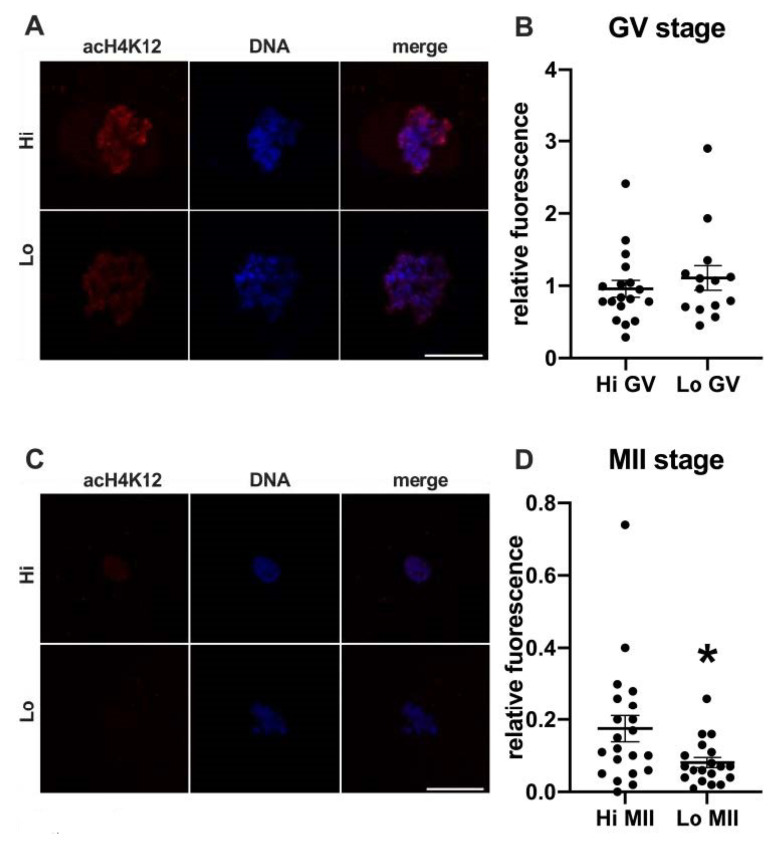
Acetylation of histone H4K12 (acH4K12) in oocytes collected from Hi and Lo ovaries. (**A**,**C**) Representative images of the chromatin of GV oocytes (**A**) and MII oocytes (**C**) stained for acH4K12 (red) and DNA (blue) and merged. Scale bar = 10 μm. (**B**,**D**) The dot blots represent the relative fluorescence of acH4K12 normalized by the DNA signal in GV (**B**) and MII oocytes (**D**). Each dot represents one oocyte. The mean ± s.e.m. is also indicated. * *p* < 0.05, Mann–Whitney test.

In this study, we examined the acetylation of H4K5 and H4K12 by indirect immunofluorescence (Figure 1 and Figure 2, respectively) and quantified the relative fluorescence in 70 oocytes collected from Hi ovaries and 61 oocytes collected from Lo ovaries (Hi- and Lo-oocyte, respectively) at the GV and MII stages. Consistent with previous reports [67], we observed general deacetylation of MII oocytes compared to the GV stage. Although extremely faint, the acetylation level in Hi-MII oocytes was consistently higher than that in the Lo counterpart, where it completely disappeared, both for H4K5 and H4K12 (*p* < 0.05, Figure 1C,D and Figure 2C,D).

Conversely, we did not observe differences at the GV stage (Figure 1A,B and (Figure 2A,B), likely suggesting that alterations in the expression/activity of enzymes responsible for acetylating–deacetylating H4 occur during oocyte maturation.

#### 3.1.2. Phosphorylation of the Histone Variant H2AX (H2AXγ)

**Figure 3:**
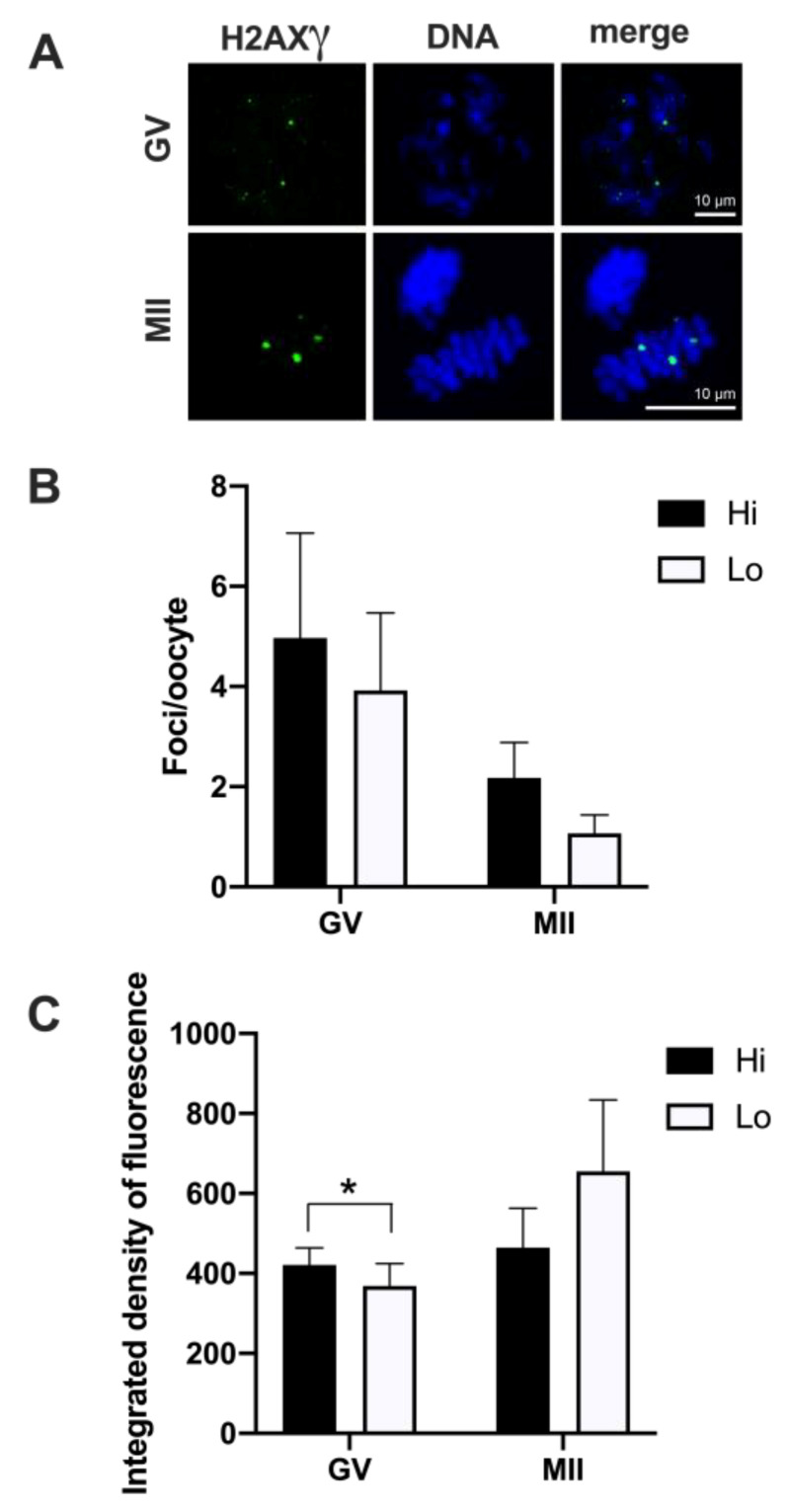
Phosphorylation of histone H2AX (H2AXγ) in oocytes collected from Hi and Lo ovaries. (**A**) Representative images of the chromatin of GV and MII oocytes stained for H2AXγ (green) and DNA (blue) and merged. Scale bar = 10 μm. (**B**) The bar graph represents the mean number ± s.e.m. of H2AXγ foci per oocyte at the GV and MII stages. Data were analyzed by Mann–Whitney test. (**C**) The bar graph represents the mean integrated density ± s.e.m. of H2AXγ foci at the GV and MII stages collected from Hi and Lo ovaries. * *p* < 0.05, Mann–Whitney test.

Overall, 50 Hi- and 41 Lo-oocytes were analyzed to investigate the extent, area, and fluorescence intensity of H2AXγ foci. We did not observe differences in the mean foci number between Hi- and Lo-oocytes at any of the meiotic stages considered (Figure 3A,B).

**Figure 4:**
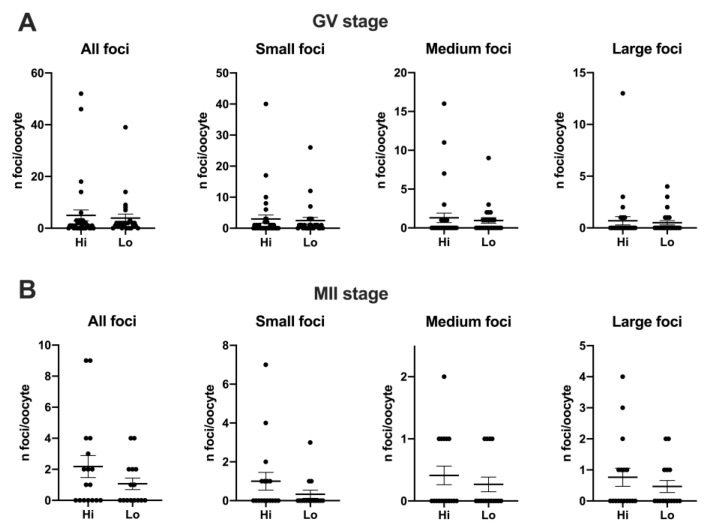
Distribution of H2AXγ foci in GV and MII oocytes collected from Hi and Lo ovaries. The dot blots represent the number and size of H2AXγ foci per GV (**A**) and MII (**B**) stage oocytes. Each dot represents one oocyte. Mean ± s.e.m. is also indicated. Data were analyzed by Mann–Whitney test.

Despite an apparent higher mean foci number at the GV stage (4.97 ± 12.02 s.d. and 3.92 ± 7.89 s.d. in Hi- and Lo-oocytes, respectively) compared to the MII stage (2.18 ± 2.92 s.d. and 1.07 ± 1.44 s.d. in Hi- and Lo-oocytes, respectively), such differences were not significant, likely due to the high inter-oocyte variations. Since we observed that the area of the single H2AXγ focus could vary considerably, we proceeded to classify the foci in small, medium, and large and count the mean foci number in each class. In this case, the mean foci number was not different for any of the size categories considered (Figure 4).

However, when we quantified the H2AXγ signal, we observed that the integrated density of the foci was higher in Hi-GV oocytes compared to the Lo-GV (Figure 3C). Since the integrated density is a function of both the area and the fluorescence intensity of the signal, we sought to measure the contribution of each parameter to the final integrated density. We observed that a higher fluorescence intensity rather than the area of the H2AXγ signal contributed to the differences in the integrated intensity when all the foci were considered, irrespective of their dimensions (Table 1—GV, Total).

We then extended the same analysis taking into account the classification in small, medium, and large foci size and observed that only the small foci class was affected. Specifically, the small H2AXγ foci of the Hi-GV oocytes showed higher fluorescence intensity and larger foci area, overall increasing the integrated density compared to the Lo-GV (Table 1—GV, small).

No differences were observed at the MII stage.

### 3.2. Cytoplasmic Maturation

#### 3.2.1. Mitochondria Distribution and Activity

**Figure 5:**
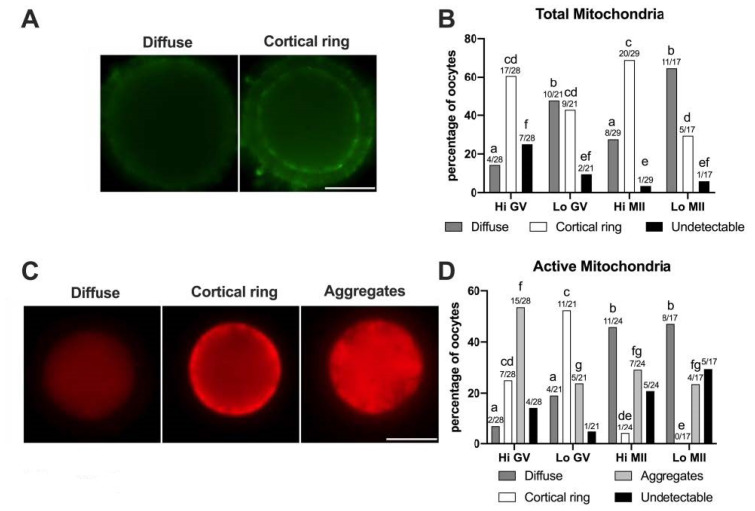
Pattern of distribution of total and active mitochondria in oocytes collected from Hi and Lo ovaries. (**A,C**) Representative images of oocytes stained with MitoTracker FM Green (total mitochondria—**A**) showing diffuse and cortical ring patterns and with MitoTracker Orange CMTMRos (active mitochondria—**C**) showing diffuse, cortical ring, and aggregate patterns. Scale bar = 50 μm. (**B**) The bar graph represents the percentage of oocytes showing diffuse or cortical ring patterns or undetectable signals. Different superscripts indicate significant differences (a,b: diffuse; c,d: cortical ring; e,f: undetectable) by Fisher’s exact test. Numbers on top of the bar indicate the number of oocytes observed for each pattern on the total oocytes observed in each meiotic stage/ovarian category. (**D**) The bar graph represents the percentage of oocytes showing diffuse, cortical ring, and aggregate patterns or undetectable signals. Different superscripts indicate significant differences (a,b: diffuse; c,d,e: cortical ring; f,g: aggregates) by Fisher’s exact test. Numbers on top of the bar indicate the number of oocytes observed for each pattern on the total oocytes observed in each meiotic stage/ovarian category.

In this study, 57 Hi- and 38 Lo-oocytes were used, at both the GV and MII stages. First, we examined the distribution of the total mitochondria using the relative fluorescence of MTG, a dye that becomes fluorescent once it accumulates in the membrane lipids of mitochondria regardless of membrane potential. As shown in Figure 5A, two classes of distribution were observed, which we named diffuse and cortical ring, similar to those reported in previous studies on bovine oocytes [49,81].

In some oocytes (3.45–25%) the MTG signal was undetectable, as also observed by Tarazona and colleagues [81], where up to 83% of GV bovine oocytes did not show an MTG staining. The perinuclear localization of mitochondria that has been described in porcine oocytes [82] was not identified in our experiments, according to the reports on bovine oocytes [49,81].

The morphological analysis of the mitochondrial distribution assessed by MTG (Figure 5B) revealed that the Hi-oocytes mainly show a cortical ring pattern (60.71% and 68.97% in GV and MII stages, respectively), as opposed to Lo-oocytes where the diffuse pattern was prevalent, in particular at the MII stage (64.71%). These results suggest that oocytes from Lo ovaries fail to allocate mitochondria in the oocyte cortex.

Next, we examined the mitochondrial distribution using MTO, a potential-sensitive, cell-permeant probe that is readily sequestered only by actively respiring mitochondria (Figure 5C,D). We observed three classes of distribution that we named diffuse, cortical ring, and aggregates, comparable to patterns described in previous reports in horses [83] and pigs [50,84]. In this case, we did not observe a perinuclear localization of MTO, as described in mouse oocytes [85]. The pattern of cytoplasmic aggregates, not observed with MTG, is significantly more frequent in Hi-oocytes at the GV stage (53.57%) than in Lo-oocytes at the same meiotic stage (23.81%, *p* < 0.05). A similar pattern of mitochondria distribution, which the authors termed ‘clumped’, was also observed in human GV oocytes [77]. Furthermore, we observed that, regardless of the ovarian category, most MII oocytes had a diffuse MTO pattern (45.83% and 47.06% for Hi-MII and Lo-MII, respectively) compared to GV oocytes (7.15% and 19.05% for Hi-GV and Lo-GV, respectively), again in agreement with findings in humans [77]. Finally, a greater percentage of oocytes showing a cortical ring (25% and 52.38% for Hi-GV and Lo-GV, respectively) was observed at the GV stage compared to MII (4.17% and 0% in Hi-MII and Lo-MII, respectively).

**Figure 6:**
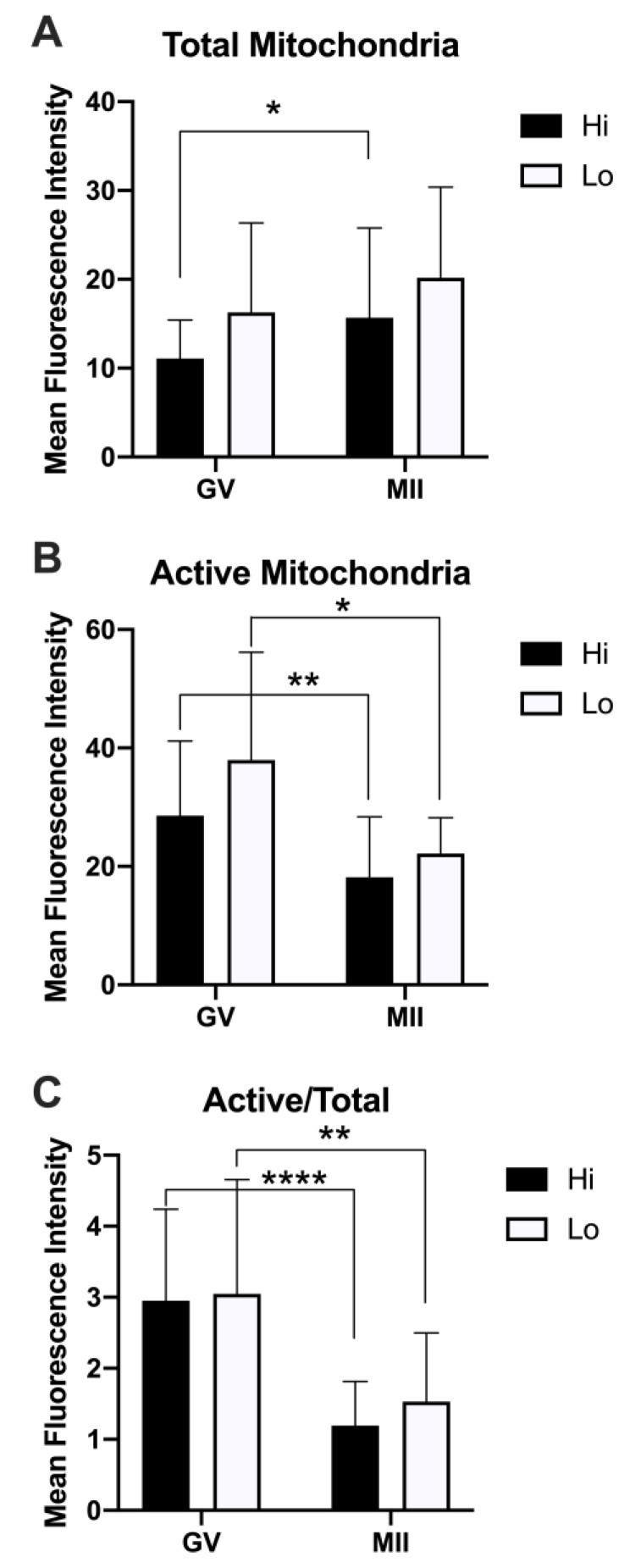
Quantification of the relative fluorescence of total and active mitochondria in oocytes collected from Hi and Lo ovaries. (**A**) The bar graph represents the mean relative fluorescence intensity ± s.e.m. of oocytes stained with MitoTracker FM Green (MTG, total mitochondria). (**B**) The bar graph represents the mean relative fluorescence intensity ± s.e.m. of MitoTracker Orange CMTMRos (MTO, active mitochondria). (**C**) The bar graph represents the ratio of the relative fluorescence intensity ± s.e.m. of the MTO/MTG (active/total mitochondria) signal. * *p* < 0.05, ** *p* < 0.01, Mann–Whitney test (**A**,**B**); ** *p* < 0.01, **** *p* < 0.0001, unpaired T-test (C).

When we analyzed the relative fluorescence of MTG and MTO as a proxy for the quantification of the total and active mitochondria, we did not observe differences between Hi- and Lo-oocytes. Rather, we observed differences when comparing the meiotic stages (Figure 6).

Specifically, the total mitochondria increased at the MII stage compared to the GV, reaching a statistical significance only in the Hi category (Figure 6A). Conversely, the mitochondrial activity was higher in GV oocytes compared to MII (Figure 6B), indicating a reduced mitochondrial membrane potential in MII oocytes, which is more evident when normalizing the MTO signal by the MTG signal (Figure 6C). Indeed, such analysis has been proposed as a relative measure of the mitochondrial activity that is independent of the mitochondrial mass [76,78].

#### 3.2.2. GSH Content and Cysteamine Supplementation

The intraoocyte GSH content was measured in 235 Hi- and 200 Lo-oocytes. Despite having similar GSH content at the time of collection from the ovary (GV stage), Hi-oocytes accumulated significantly more GSH during IVM compared to the Lo counterpart (Table 2).

Supplementation with cysteamine during IVM increased the GSH content in both Hi- and Lo-oocytes, to a similar extent.

To test whether the low intraoocyte GSH might be a determinant of the impaired developmental competence in Lo-oocytes, we devised a rescue experiment in which cysteamine was supplemented during IVM and the oocytes were then fertilized and cultured for 8 days. Overall, 430 Hi- and 397 Lo-oocytes were used in these experiments. In agreement with previous reports, the cleavage and blastocyst rates were lower in Lo-oocytes (Table 3).

However, cysteamine supplementation rescued, at least in part, the developmental competence of Lo-oocytes, as shown by an increase in blastocyst yield from 6.2 to 20.1% (blastocysts/total oocytes). Conversely, the overall quality of the blastocysts does not seem to be affected, as suggested by the similar cell numbers composing the blastocysts, irrespective of oocyte category or treatment.

### 3.3. Oocyte–Cumulus Cell Interactions

**Figure 7:**
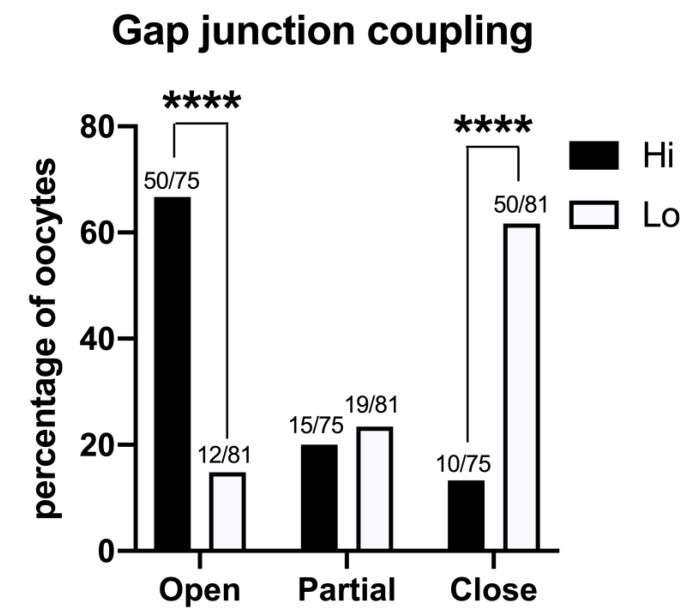
Gap junction functionality between the oocyte and cumulus cells in COCs collected from Hi and Lo ovaries. The bar graph represents the percentage of oocytes with open, partially open, and closed gap junction communications as judged by the spreading of intraoocyte-injected Lucifer yellow, a gap junction-permeant dye. **** *p* < 0.0001, Fisher’s exact test. Numbers on top of the bar indicate the number of COCs observed for GJ type on the total COCs observed in the ovarian category.

The GJ-permeant dye Lucifer yellow was injected in the ooplasm of 75 Hi- and 81 Lo-oocytes at the GV stage. The spreading of the dye to the cumulus cells indicated that the majority of the Hi-oocytes had functionally open GJs between the oocyte and the surrounding somatic cells (50/75, 66.7%); in comparison, only 14.8% (12/81) of Lo-oocytes showed open GJs (Figure 7). 

Most of the Lo-oocytes were characterized by closed GJs (50/81, 61.7%), while only 13.3% of the Hi-oocytes considered (10/65) were characterized by closed GJs (Figure 7).

## 4. Discussion

Whereas the progressive depletion of the ovarian reserve, which leads to menopause and infertility, occurs in the fifth decade of life, women experience a fertility decline as early as in their mid-30s [1], hence considerably earlier compared to the onset of menopause. Since aging patients can still carry a pregnancy to term with oocyte donation and embryo transfer, a decline in oocyte quality is regarded as the major cause of age-associated infertility [3].

Due to the growing impact that reproductive aging has on society and health care policy, reproductive scientists have focused on developing animal models to experimentally address the ovarian aging problem. Accordingly, such animal models must have a short life cycle to enable research on key phases of the ‘aging spectrum’. Another approach is to induce aging-like phenotypes by genetically manipulating the animals or to use aging-like models that generate spontaneously but need to be extensively characterized. In this sense, it has been observed that human and bovine ovaries consistently showing a low AFC share many similarities with the ovaries undergoing normal reproductive aging. Low-AFC cow ovaries were therefore proposed as a model to study ‘early’ ovarian aging [21,40,43].

The bovine model of low-AFC ovary was described in the late 1990s, and during the years it has been morphologically and biochemically characterized at the organ level. However an in-depth characterization of the ‘quality’ of the gametes has not been conducted thus far, and investigations were confined to observing low blastocyst development [22,39,42] and high incidence of aneuploidy [44]. In the present study, we confirm and expand previous findings. Specifically, we observed alterations encompassing epigenetic modifications, organelle allocation, redox ability, and oocyte–cumulus cell communications, in line with the hypothesis of a multifactorial failure of the maturation process. Notably, by rescuing one of the considered parameters, we also provide a means for improving the decreased oocyte quality.

In the first set of experiments, we investigated the global acetylation level of histone H4, since the residue K12 in particular has been linked to the phenotype of increased aneuploidy during physiological aging in mouse oocytes [51,52,53]. Specifically, these studies reported higher residual acetylation in eggs retrieved from aged mice. Conversely, we found that Lo-MII oocytes show excessive deacetylation at both K12 and K5, more in line with the findings that we previously reported in another mono-ovulatory large mammal. A correlation was in fact shown in horse oocytes between severe deacetylation and aneuploidy/spindle defects [86,87]. Whether these discrepancies are species-specific or due to other experimental conditions (IVM vs. superovulation; physiological aging vs. ‘early aging-like’ phenotype) remains to be addressed. However, in our studies, both Lo- and Hi-MII oocytes were obtained by IVM, seemingly indicating that alterations in the epigenetic remodeling can impact the process of chromosome segregation during oocyte maturation. Although a mechanistic explanation linking the global H4 acetylation levels and aneuploidy is still lacking, we propose the hypothesis that the levels of global histone acetylation might affect the association between chromosomes and other factors necessary for proper segregation.

A study conducted in the mouse also reports differences in the acetylation of some K residues at the GV stage [52]. However, we only found differences at the MII stage, suggesting that alterations in the expression or activity of enzymes responsible for acetylating–deacetylating H4 occur during oocyte maturation and are not evident in the prophase I oocytes.

Other histone modifications have been implicated in genomic instability and cell senescence [88]. Among them, phosphorylation of the histone variant H2AX in the position of Ser139, known as H2AXγ, is triggered by the formation of double-strand breaks (DSBs) in the DNA [89]. Programmed DSBs are generated at the beginning of meiotic recombination and represent a stage in the formation of synapses between homologous chromosomes [90,91]. However, persistent unrepaired or newly formed DSBs produced by oxidative damage or exogenous factors are genotoxic. Therefore, they represent potential sources of genomic instability and chromosome aberrations [92,93].

In our experiments, we observed a high incidence of H2AXγ foci irrespective of the ovary type and meiotic stage. The highest source of variability seemed to be the single oocyte itself, with some gametes carrying tens of foci and others having none or few. Since just one unrepaired DSB has potentially lethal consequences, it was quite surprising to find that 71% of oocytes at the prophase I stage (42/59 GV oocytes overall) showed H2AXγ foci. Nevertheless, they must still be able to progress through meiosis and reach the MII stage, given that the maturation rate is approximately 80–90%. It seems therefore that bovine oocytes can escape the arrest of cell cycle progression that affects other cell types when the DNA damage response (DDR) complex gets activated (recently reviewed in [94]). This finding is in agreement with previous studies conducted in mouse oocytes [95]. Furthermore, we found that the integrated density of the H2AXγ signal was higher in Hi-oocytes at the GV stage. This last observation seems to suggest that Hi-oocytes might be more efficient in signaling the DNA damage and that H2AXγ might serve the purpose of more efficiently recruiting the DDR complex at the site of DSB for the initiation of DNA repair. However, this hypothesis does not seem to be supported by the finding that no differences were seen at the MII stage in H2AXγ foci abundance, area, and signal intensity. One intriguing possibility is that the differences in the H2AXγ signal intensity that we observed at the GV stage derive from a different accessibility of the GV chromatin to the antibody in the two oocyte-types (Franciosi F. personal observation). This hypothesis is currently under investigation.

At the level of cytoplasmic maturation, changes in mitochondrial distribution might be linked to the phenotype of increased aneuploidy and decreased developmental competence. Mitochondria not only produce most of the ATP necessary for the cell via oxidative phosphorylation (OXPHOS) but also are involved in apoptosis, calcium homeostasis, thermogenesis, and gene expression [96]. In addition, reactive oxygen species (ROS) generated in the mitochondria seem to have key physiological signaling functions during early development [97].

Distinct patterns of mitochondrial distribution have been described at different stages of oocyte development. Since defects in mitochondria distribution have been shown to produce a disproportionate segregation between the blastomeres that negatively affects embryo development [98], the defect in allocation that we observed might explain, at least in part, the decreased embryo development obtained with Lo-oocytes. Other reports showed that an insufficient number of mitochondria negatively impacts the oocyte developmental competence [99]. However, it does not seem to be the case in Lo-oocytes, where a decrease in total mitochondria compared to age-matched control was not observed.

Higher mitochondrial activity has been associated in some studies with meiosis progression [81,83,84] and developmental potential [77,78]. We did not observe such a trend but did observe a general decrease in activity during meiosis. Furthermore, no differences were evident between Hi and Lo ovarian categories. Indeed our observations seem compatible with other reports of low oxygen consumption and low ATP production in the oocyte and early embryos [100] and with the use of the ‘adenosine salvage pathway’, described in the bovine oocyte, as an alternate method to produce ATP and meet the energy demands [101].

Finally, we would like to draw attention to the fact that we observed different patterns of distribution of total and active mitochondria within the same oocyte. Although initially confusing, this might simply indicate that mitochondria with low ATP production are displaced in cellular compartments where they fulfill functions other than energy production [102].

Living cells are subjected to free radicals that are produced by the cell metabolic processes and can induce severe injuries [103]. Scavengers of free radicals are therefore essential for cell survival since they control the damaging effects of these highly reactive molecules. GSH is the major nonprotein sulfhydryl compound involved in the maintenance and regulation of the thiol redox status, thus protecting the cell from oxidative damage [104,105]. As oocytes are long-living cells, cumulative insults from oxidative damage are likely to accumulate more than in other cell types. Therefore, in the present study, we included experiments to investigate GSH, representing one of the key molecules of the redox status ‘surveillance system’. Indeed a role of the GSH ooplasmic reservoir in protecting the zygote and the early embryos from oxidative damage has been already hypothesized [56,57]. Furthermore, GSH has been shown to promote the formation of the male pronucleus upon fertilization [106]. Our findings align with the notion that the ooplasmic GSH content is an important component of cytoplasmic maturation and developmental competence. We observed not only that Lo-oocytes fail to accumulate GSH during maturation, but also that increasing the GSH content via cysteamine supplementation of the IVM medium rescues, at least in part, the ability of Lo-oocytes to form blastocysts.

Cysteamine supplementation promotes the uptake of cysteine, a limiting molecule in the GSH synthesis [107], and it has been shown to increase intracellular GSH content in bovine oocytes, as well as in other species (reviewed in [108]). Besides demonstrating that a low GSH content impairs the quality of Lo-oocytes, our experiments provide a proof of concept that in-depth molecular characterization sustains the development of strategies to overcome cellular defects.

The GSH content of an oocyte is highly correlated with the presence of cumulus cells and functional GJs [109]. Furthermore, there are countless reports of the beneficial effects of the cumulus cell–oocyte interactions in order to promote developmental competence, as reviewed in [110,111]. In this context, the assessment of GJ functionality in Hi- and Lo-oocytes confirms that gametes showing a higher rate of closed GJs are also affected by low blastocyst rate and low GSH content.

Overall, we observed several deficiencies in Lo-oocytes, encompassing diverse aspects of the nuclear and cytoplasmic maturation and in the interactions with the follicular environment. Our study seems to indicate that multiple defects concur in determining a decrease in the oocyte developmental competence and an increased incidence of aneuploidy in a model of early ovarian aging, suggesting a multifactorial etiology. This observation is in agreement with the literature on reproductive aging, which reports multiple defects associated with aneuploidy in old oocytes, such as DNA damages [112,113], epigenetic changes [51,52], malfunction of spindle assembly checkpoints [5,114], oxidative and mitochondrial stress [115,116], and shortened telomeres [117], just to name some.

Some of the findings that we report here are still preliminary observations and deserve to be investigated further. Furthermore, whether the defects are intrinsic to the oocyte and affect its ability to interact with the environment or derive from an altered follicular environment and a lack of oxygen and nutrients caused by the compact ovarian stroma and limited vascularization of the theca still waits to be addressed [42]. However, the GSH content seems to be a key factor affecting the developmental competence in Lo-oocytes. The possibility of increasing the ooplasmic GSH content by supplementing the medium with cysteamine seems to be a promising option for treating aging-related deficiencies in embryo development. It is not excluded that other free radical scavengers might also promote the maturation of an oocyte of better quality.

A better comprehension of the mechanisms involved in the onset of reproductive failure both in early and physiological aging will improve the possibility of treating in a more targeted way or preventing this type of infertility. For instance, finding markers for early diagnosis of POF might indicate the need for social egg/embryo cryopreservation before the ovarian reserve and oocyte quality are impaired.

## Figures and Tables

**Table 1:** Area, fluorescence intensity, and integrated density of H2AXγ foci in GV and MII oocytes from Hi and Lo ovaries.

Nuclear Stage	Group		Area (µm^2^)	Fluorescence Intensity	Integrated Density
GV	Total	Hi	0.14 ± 0.01	2799 ± 27.09 a	421.6 ± 42.35 a
		Lo	0.13 ± 0.02	2652 ± 36.24 b	368.9 ± 55.31 b
	Small	Hi	0.046 ± 0.002 a	2616 ± 23.82 a	121.9 ± 6.94 a
		Lo	0.039 ± 0.002 b	2486 ± 24.93 b	98.9 ± 7.48 b
	Medium	Hi	0.17 ± 0.01	2941 ± 32.86	510.9 ± 28.2
		Lo	0.16 ± 0.01	2800 ± 73.13	445 ± 41.75
	Large	Hi	0.43 ± 0.03	3277 ± 63.56	1443 ± 131.2
		Lo	0.53 ± 0.07	3187 ± 106.6	1551 ± 215.6
MII	Total	Hi	0.27 ± 0.04	1429 ± 52.15	464.5 ± 98.21
		Lo	0.36 ± 0.08	1553 ± 99.44	655 ± 178.9
	Small	Hi	0.04 ± 0.01	1227 ± 35.26	49.15 ± 6.04
		Lo	0.04 ± 0.01	1231 ± 57.2	55.48 ± 20.06
	Medium	Hi	0.18 ± 0.02	1396 ± 76.82	250.8 ± 36.65
		Lo	0.22 ± 0.05	1409 ±113.8	322.4 ± 93.19
	Large	Hi	0.62 ± 0.06	1711 ± 91.39	1123 ± 158
		Lo	0.61 ± 0.18	1809 ± 141.8	1154 ± 249.9

Values are expressed as mean ± s.e.m. a and b indicate *p* < 0.05, Mann–Whitney test.

**Table 2:** Intraoocyte GSH content (pmol/oocyte) of oocytes isolated from Lo and Hi ovaries in vitro matured with and without cysteamine 100 µM.

	N	GV	N	MII
Hi	85	4.51 ± 0.42a	65	6.59 ± 0.39b
Hi + Cyst	-	-	85	10.45 ± 0.88c
Lo	65	4.31 ± 0.41a	55	4.36 ± 0.31a
Lo + Cyst	-	-	80	9.88 ± 0.77c

Values are expressed as mean ± s.e.m. a, b, and c indicate *p* < 0.05, one-way ANOVA followed by Tukey’s multiple comparison test.

**Table 3:** Effect of cysteamine administration during maturation on blastocyst development of COCs isolated from Lo and Hi ovaries.

Treatments	Total Oocytes	% Cleaved	% Blastocysts on Cleaved	% Blastocysts on Total	Cell Number
Hi	212	94.9 ± 0.5b	36.1 ± 2.6c	34.2 ± 2.4c	88.9 ± 5.4
Hi + Cyst	218	96.4 ± 1.2b	34.6 ± 3.9bc	33.3 ± 3.8c	99.8 ± 5.8
Lo	201	86.9 ± 1.3a	7.2 ± 1.9a	6.2 ± 1.6a	84.9 ± 6.9
Lo + Cyst	196	84.3 ± 3.1a	24.2 ± 3.8b	20.1 ± 2.9b	96.5 ± 6.2

Values are expressed as mean ± s.e.m. a, b, and c indicate *p* < 0.05 within columns, one-way ANOVA followed by Tukey’s multiple comparison test.

## Data Availability

Data are contained within the article.
